# COVID-19 herd immunity in the absence of a vaccine: an irresponsible approach

**DOI:** 10.4178/epih.e2021012

**Published:** 2021-02-03

**Authors:** Jade Khalife, Derrick VanGennep

**Affiliations:** 1 Social Medicine and Global Health, Faculty of Medicine, Lund University, Malmö, Sweden; 2 New England Complex Systems Institute, Cambridge, MA, USA

**Keywords:** Herd immunity, Vaccines, Coronavirus, Ethics, Endemic diseases

## Abstract

As severe acute respiratory syndrome coronavirus 2 (SARS-CoV-2) continues to spread rapidly throughout the human population, the concept of “herd immunity” has attracted the attention of both decision-makers and the general public. In the absence of a vaccine, this entails that a large proportion of the population will be infected to develop immunity that would limit the severity and/or extent of subsequent outbreaks. We argue that adopting such an approach should be avoided for several reasons. There are significant uncertainties about whether achieving herd immunity is possible. If possible, achieving herd immunity would impose a large burden on society. There are gaps in protection, making it difficult to shield the vulnerable. It would defeat the purpose of avoiding harm caused by the virus. Lastly, dozens of countries are showing that containment is possible.

## WHAT IS HERD IMMUNITY?

Herd immunity is reached when a sufficiently large proportion of a population has become immune to infection, not only protecting themselves, but also decreasing the likelihood of transmission of disease to remaining susceptible persons. Immune persons thus form a barrier to slow or prevent the disease outbreak among other members of the “herd.”

The critical proportion of a population (p_c_) needed to be immune to a disease before herd immunity becomes protective is roughly estimated using the basic reproductive number (R_0_) of the disease as follows:

$$p_{c}\;=\;1-\frac{1}{R_{0}}$$

R_0_ is an average that varies by factors such as population density, age structure, individual behaviors, and social interactions. For coronavirus disease 2019 (COVID-19), R_0_ varies globally, but typically is about 3.0 [1], which means that we would need roughly 67% of the population to be immune. In the absence of a vaccine or pre-existing immunity, this means allowing two-thirds of the population to be infected, which can come with dire consequences.

In this paper, we describe why a strategy that aims to reach herd immunity against COVID-19 in the absence of a vaccine is deeply irresponsible.

### It results in a large loss of life, more severe disease, and long-term harm

The COVID-19 infection fatality ratio (IFR) varies by population age, comorbidities, healthcare access, and other factors, but can be expected to range between 0.37% and 1.45% [2]. This would make COVID-19 at least 10 times worse than seasonal influenza, meaning that reaching 67% infection would roughly translate into about 24,790 to 97,150 deaths in a population of 10 million people—for their first infection only. This provides an idea of the scale of deaths involved. However, in the real world, the spread of the disease would not instantly stop once 67% of persons are infected; instead, disease spread would continue further and over-shoot to some extent. More lives would be lost if the healthcare system is overwhelmed by having to care for over a million persons who would present with severe or critical disease due to COVID-19 [3].

Another consequence of such an approach would be accepting that many, if not most, survivors would develop persistent symptoms and chronic conditions. Evidence is already accumulating of chronic damage to the lungs, brain, heart, kidneys, and other organs and body systems due to COVID-19 [4-8]. This largely neglected burden remains uncaptured in national and international tallies. The patient-created term “long COVID” has been used for long-term sequelae caused by the disease, such as fever, fatigue, headache, loss of smell/taste, myalgia, and shortness of breath. Up to 1 in 5 of COVID-positive persons reported such symptoms for 5 weeks or longer, while 1 in 10 reported them for 12 weeks or longer, with a median of 40 days [9]. Even among those with mild or initially asymptomatic infections, 21% reported symptoms for 30 days or longer after infection [10]. COVID-19 is more accurately considered to be a complex and multi-system disorder, rather than a respiratory disease [11], with a long-term impact certain to contribute to the global burden of various diseases.

### It results in endemic disease, not the absence of disease, with ongoing harm

Herd immunity, if at all reachable, is not a one-time achievement, but has to be maintained through new infections (and consequently deaths). This is largely due to 2 aspects: population flux and duration of functional immunity. With populations constantly in flux due to births, deaths, and migration, new infections must continually occur to maintain the protective threshold. The immunity duration for COVID-19 is uncertain, possibly ranging from a few months to a few years. For severe acute respiratory syndrome coronavirus 1 (SARS-CoV-1, in the 2002-2004 outbreak) immunity typically lasted 2 years [12], and for seasonal coronaviruses only 6-12 months [13]. We know that COVID-19 reinfection is possible, following the confirmation of a few persons who were infected a second time a few months after their initial infection [14,15]. However, until wider investigations are conducted, the extent and duration of immunity remains uncertain.

Maintaining herd immunity with new infections would also have to occur at a rate that avoids overwhelming the healthcare system, otherwise death rates could increase considerably. This also means that some physical distancing measures would likely become permanent, together with the associated costs to society.

Herd immunity would also be a misleading term in the absence of a vaccine. It suggests that a community has become immune, whereas in reality the disease would have become endemic and many people would still continue to be infected.

### Protecting large vulnerable groups becomes nearly impossible, as they cannot safely participate in society where disease is endemic

Herd immunity functions at the level of the population and of surrounding contacts. If more than two-thirds of a population is immune to COVID-19 (due to previous infection), this may be protective for the remaining non-immune persons. However, no population is completely homogeneous. Persons with contacts who are largely not immune would form unprotected pockets vulnerable to outbreaks. This particularly applies to elderly individuals in nursing homes, as well as remote/rural/religious/other communities that are to some extent less inter-mixed with the population majority or have been less exposed to the disease.

### Reinfection undermines immunity, especially because the disease can be more severe in subsequent infections

In general, persons reinfected with COVID-19 would be expected to have a less severe course than their previous infection, due to the presence of long-term memory cells. However, exceptions exist where viral reinfection triggers a reaction worse than the initial infection, as in dengue fever [16]. This also seems to be the case in 8 of the 24 COVID-19 reinfections confirmed to date [17]. It is also relevant to consider that some COVID-19 survivors may have more severe or fatal reinfections due to chronic damage sustained from their previous infection. This further contributes to the list of uncertainties.

It is relevant to distinguish between functional immunity, which protects from illness, and the body’s immune response. Recent research has found that antibody levels for COVID-19, made by B lymphocyte cells, steeply decline only 2-3 months following infection [18,19]. This pattern is typical of the immune response to viral threats. The harder-to-detect T lymphocyte cells (e.g. helper, cytotoxic, memory T-cells) typically have a greater role in longer-term immunity than antibody levels, as do B lymphocytes themselves. A preliminary study found that while some T-cells decreased within a few months of infection, B-cells remained stable up to 6 months after infection [20]. However, we currently lack sufficient knowledge on how this translates to functional immunity to COVID-19.

It is also unknown whether the recently discovered cross-reactivity of T-cells from other coronaviruses (e.g., previous common cold infection) would confer any advantage to persons infected with severe acute respiratory syndrome coronavirus 2 (SARS-CoV-2, which causes COVID-19) [21-23]. If it does, this would be in the form of reduced severity or speed of severity progression, but it would not impact infection, as T-cells are activated after viral replication within cells has already occurred (i.e., after infection). It is also uncertain whether such cross-reactivity may actually worsen the outcome of severe COVID-19 in some individuals [24].

### Containing the virus has been successful in dozens of countries, while attempting herd immunity has failed with a high cost

Approximately 30 countries are succeeding at containment of COVID-19, with zero or near-zero daily cases [25]. These include large populations in low/middle-income countries such as Cambodia, Laos, Mongolia, Thailand, and Vietnam, as well as Australia, China, New Zealand, Singapore, and Taiwan.

**Figure 1.**
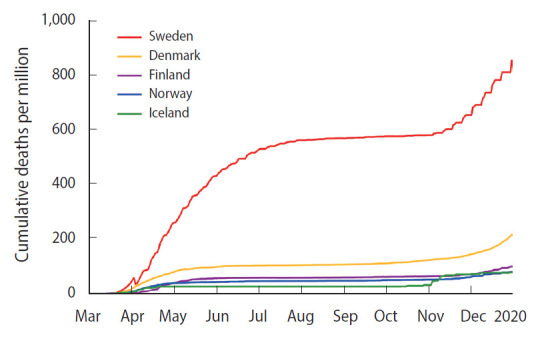
Cumulative deaths per million from COVID-19 among Nordic countries [27].

Some countries initially chose to aim towards herd immunity, either implicitly or explicitly, such as the United States, United Kingdom, and the Netherlands. Sweden stands out as a country that has maintained its goal of reaching herd immunity, adopting limited measures intended to slow, but not stop, the spread of disease. With about 9,812 deaths by the end of December 2020 [26], the mortality rate from COVID-19 in Sweden has been several times that of its neighbors Denmark, Finland, Iceland, and Norway (Figure 1). Sweden’s mortality rate is among the highest worldwide, and Sweden is currently seeing a rapid resurgence of cases, with no direct evidence suggesting that herd immunity is near. Sweden’s Nordic neighbors are currently experiencing modest caseloads compared to Sweden.

One may counter that this approach allows a high death toll early on, while building immunity to lower the toll later. However, this ignores that the most important endpoint is every life lost, not an arbitrary point in time. Experimental clinical trials are ended once the intervention arm has a mortality rate that exceeds that of the control. Such ethical standards were developed to safeguard humans from unethical experimentation. If the Swedish approach were a clinical trial, it would have ceased long ago when the large difference in mortality became apparent.

The largely uncontrolled outbreak in Manaus, Brazil is also instructive. The first outbreak peaked in Manaus in April 2020, followed by several months of relatively low and stable spread. Estimates suggest that by October, about 76% of the population had been infected [28]. However, a resurgence began in December 2020, which at the time of writing remains ongoing. Four potentially overlapping explanations have been suggested: overestimation of the first surge’s attack rate, waning immunity, new viral lineages evading immunity generated from previous infection, and higher transmissibility of new lineages [29]. Even with the relatively low IFR (0.17-0.28%) in Manaus [28], the loss of life has had a devastating impact.

### Suppressing the disease gives time for the development of treatments and vaccines

The time gained by suppressing transmission is valuable, as it allows the development of more effective medications and treatment approaches for COVID-19 cases. Survival outcomes of COVID-19 have already improved since the early months of the outbreak due to such developments, including the use of anticoagulant therapy and systemic corticosteroids for severe or critical cases. Other trials are also underway, such as those for monoclonal antibodies. As such, arguments that a herd immunity approach simply involves up-fronting the costs (or deaths) are misleading.

### It defeats the purpose of reducing harm caused by the virus

Aiming at herd immunity in the absence of a vaccine for a disease with a relatively high mortality rate such as COVID-19 defeats the purpose of this public health approach, namely, to save lives. A strategy that allows people to be infected by a disease is not one that protects them from the disease. While preventing healthcare systems from being overwhelmed is important, it is the system that serves the individual, not the other way around.

Protecting the most vulnerable members of society is a defining feature of humanity. Allowing their exposure to the great harm posed by such a herd immunity approach makes this not only an unscientific gamble, but also highly unethical.

### There are various uncertainties associated with a novel virus

An approach aiming at herd immunity without using a vaccine involves several uncertainties, including uncertainty regarding the extent and duration of immunity, severity of reinfection, role of cross-reactivity, persistent symptoms, and chronic conditions. Another aspect to consider is that every infected person is a laboratory for potential new viral strains to emerge through mutation and recombination. Recently, a new strain (lineage B.1.1.7) was first identified in the United Kingdom, with preliminary reports suggesting it could be up to 70% more transmissible than previous strains Allowing large numbers of people to be continually infected means keeping a wider door open for new viral strains.

Animals also pose a significant risk. Recently, more than 200 human cases of COVID-19 have been identified in Denmark with SARS-CoV-2 variants associated with farmed minks, including 12 cases with a unique variant [30]. Denmark, the Netherlands, Spain, Sweden, Italy, and the United States have all reported SARS-CoV-2 in farmed minks [30]. It is possible that the virus could also cross to other similar animals such as mice, voles, rats, and ferrets, providing more opportunities for it to evolve and cross back to humans. Providing more such opportunities for the virus among both humans and animals would therefore be unwise.

Towards the end of 2020 there has been much news coming from a few clinical trials of vaccine candidates. However, in most countries, vaccination is unlikely to reach the majority of the public until mid- to late 2021. Importantly, these vaccines could be a powerful tool to help eliminate the virus, but the timeline is too lengthy to justify only waiting for vaccination. Even under optimal conditions, vaccine efficacy will not be 100%; not all persons vaccinated will be protected. In a real-world environment, further barriers to effectiveness will also be encountered, including availability and uptake by populations. In many countries, it may be years before herd immunity through vaccination is reached. Furthermore, since immunity duration is still uncertain, the possibility remains that protection may be short-lived, thereby limiting the effectiveness of vaccination. Improved mechanisms for surveillance, monitoring, response, and treatment will remain necessary beyond vaccination.

## CONCLUSION

The greatest benefit for populations in both health and economic terms lies in containing and pursuing elimination of COVID-19. Over 30 countries are showing us how. Vaccination will further protect populations from its re-emergence. However, pursuing herd immunity without a vaccine involves numerous uncertainties, is costly in terms of lives and disease, is ineffective, and—being unethical and uncompassionate—is not compatible with human dignity and development.

### Ethics statement

Not applicable as the manuscript did not involve any experimentation.
